# Effectiveness of a flipped classroom for undergraduate in implant dentistry hands-on course

**DOI:** 10.1186/s12909-024-05536-6

**Published:** 2024-05-15

**Authors:** Tao Wu, Haibin Xia, Wei Sun, Yan Ge, Chun Liu, Fengxiao He, Tiange Cheng, Yi Zhao, Si Chen

**Affiliations:** 1 State Key Laboratory of Oral & Maxillofacial Reconstruction and Regeneration, Key Laboratory of Oral Biomedicine Ministry of Education, Hubei Key Laboratory of Stomatology, School & Hospital of Stomatology, Wuhan University, 237 Luoyu Road, Wuhan, 430079 P. R. China; 2 Center for Prosthodontics and Implant Dentistry, Optics Valley Branch, School and Hospital of Stomatology, Wuhan University, Wuhan, 430079 P. R. China; 3 Department of Oral Implantology, School &Hospital of Stomatology, Wuhan University, Wuhan, 430079 P.R. China; 4 Department of Prosthodontics, School &Hospital of Stomatology, Wuhan University, Wuhan, 430079 P. R. China

**Keywords:** Implant dentistry, Hands-on course, Flipped classroom, Teaching methods

## Abstract

**Purpose:**

The purpose of this study was to compare the learning in the implant dentistry hands-on course to that of the flipped classroom (FC) and the traditional lecture cohorts (control).

**Materials and methods:**

In this study,80 students were enrolled for the first time in an implant dentistry program. Subsequently, they were divided into two groups. The first, the FC group, which had free access to a video with a PowerPoint presentation on the Chaoxing-WHU-MOOC platform about the implant placement on first molar sites before class. The second, the control group, which attended a didactic lecture describing implant practice on the first molar site via a bidirectional multimedia interactive teaching demonstration and then operated on a simulation model. Cone beam computed tomography (CBCT) and the deviation gauge were utilized to analyze the accuracy of the implant placement in the students’ models. An online satisfaction questionnaire was distributed to both groups one week after the class.

**Results:**

The linear deviation of the CBCT examination did not show any statistical difference between the two groups concerning cervical, apex, and angular. A significant buccal deviation was observed in the control group compared with the FC group (mean: 0.7436 mm vs. 0.2875 mm, *p* = 0.0035), according to the restoration-level deviation gauge. A total of 74.36% of students in the FC group placed implant within 0.5 mm buccal-to-lingual deviations, but only 41.03% of students in the control group reached within 0.5 mm buccal-to-lingual deviation ranges. Additionally, 91.67% of the students in the FC group and 97.5% of the students in the control group were satisfied with the practical implant class.

**Conclusion:**

FC was more effective than a didactic lecture for implant dentistry practical skill acquisition.

## Introduction

Teaching methodology in dentistry faces significant challenges because of the advancement of media technologies. Students must develop abilities in critical thinking and problems-solving abilities independently. Instead of holding classroom lectures and having students apply the content in homework, students prereview the material (e.g., audiovisual presentations, videos, and websites, etc.) at home, and then have small-group discussions occur in the classroom resulting from flipped classroom (FC) learning [1–5]. The primary goal of FC is to shift learning from an instructor-centered model to a learner-centered model involving individual or team-based collaborative learning [4]. In the last two decades, FC has been introduced in different fields of dentistry, such as periodontics [3], dental anatomy [6], orthodontics [7, 8], paediatric dentistry [9], maxillofacial surgery [10], prosthodontics [11, 12], dental local anaesthetic [13]. However, it still has few applications in implant dentistry, especially in terms of hands-on learning.

As an emerging independent branch of dental, implant dentistry has advanced in education and practice over generations [14]. This course requires a combination of theory and practice, specifically students’ clinical and practical skills training. A study included 1015 respondents from 84 countries found that didactic lectures or theory-based training were the most common [14]. In recent years, the teaching mode of theoretical courses has evolved from the traditional way to a blended teaching model such as problem-based learning (PBL) and case-based learning (CBL), and various online applications have been added [15]. However, few studies have been reported about teaching method innovation in implant dentistry. It also determined that the most frequent challenge was the “identification of implant position” [14]. An overview of the U.S. predoctoral dental implant program established that 90.4% would conduct simulation exercises without direct patient care [16]. In China, students take oral implant placement hands-on classes on simulation models before attending a senior undergraduate implant teaching in their fourth or fifth years. This kind of hands-on course enables unskilled students to grasp anatomy characteristics and operation skills comprehensively for future safe clinical practice [17]. Traditionally, the teacher first demonstrates face-to-face how to perform the implant surgery on a simulation model, and then the students operate independently. Due to venue and time constraints, students may ignore the details of the operation in the short class time, and some students have poor learning initiative and lack experience. Besides, students lack initiative and a sense of self-inquiry. Therefore, a reform of the existing teaching methodology is necessary.

The combination of implantology theory and practical skill is a significant challenge; thus, exploring implant dentistry practice teaching methods is essential. However, no study in the literature has been conducted on the FC approach to implant dentistry practical skill teaching. We hypothesize that the implementation of the FC teaching method will improve students’ performances in implant dentistry practical skills. This study aimed to compare students’ learning in implant placement practice classes between an FC and a TL cohort, and assess the effectiveness of the FC methodology in implant dentistry hands-on course.

## Materials and methods

### Student recruitment

The trial involved 80 undergraduates in their fourth year at Wuhan University from November 2022 to April 2023. Inclusion was achieved through volunteering, and exclusion involved refusal to participate. Two cohorts of participants (*N* = 80 in total, *N* = 40 per group) were randomly allocated to the FC cohort and the TL cohort to complete the oral implant practice course (surgical implantation at the mandibular molar). Informed consent was obtained from all the participants. This study was approved by the Ethics Committee at the School and Hospital of Stomatology, Wuhan University (No. [2022] B73).

### Interventions

The interventions were as follows:

The subjects included in this pedagogical study had previously completed theoretical teaching related to oral implantology. The FC group had access to pre-class videos and a related PowerPoint of an implant surgery operation on Chaoxing-WHU-MOOC platform online one week before class. After the self-study, students summarized the operation’s key points and collected the problems and difficulties. During the hands-on practice, the teacher answered common questions at the beginning of the course, and students discussed in small groups (eight persons per group). Then, students operated on a simulation model independently for 90 min (two people per implant machine). One week after the class, a satisfaction questionnaire was taken online.

The control group attended a didactic lecture about the ideal management of implant practice on the first molar site via a 30-minute bidirectional multimedia interactive teaching demonstration, and then operated on the simulation model independently for two hours (two persons per implant machine). One week after the class, a satisfaction questionnaire was taken online.

### CBCT matching

A file containing an ideal implant 3D position design file was imported into the implant navigation design software, and the teacher performed the implant placement on the posterior mandibular simulation model under navigational guidance. This model was used as a standard reference model. For the two different teaching approaches, a CBCT examination of models of students placing implants was taken; and then matched with the CBCT file of the standard model to analyze the linear deviations.

### Restoration deviation gauge

**Fig. 1:**
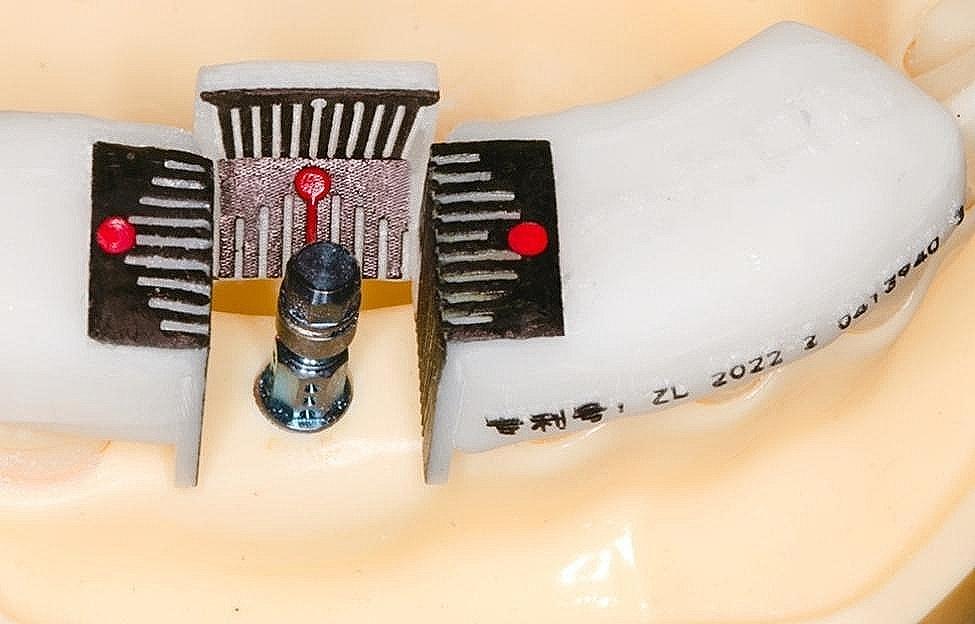
Restoration deviation measuring gauge (the red point refers to the ideal restoration position)

In addition to detecting deviations of the implants in the simulated bone with CBCT, a novel gauge was invented to analyze deviations in the penetration position of the dental implant restorations (Fig. 1). After implant placement, an implant carrier was inserted into the implant to check buccal-lingual, mesial-distal, and coronal-root depth deviations.

### Satisfaction questionnaire

A satisfaction questionnaire was developed so that the students could evaluate the clinical practice skill process one week after class (Table 1). The first question was “What is your overall feeling about this practical implant class?”, which intended to evaluate students’ overall impression of the teaching approach. The second question, “What do you think about the teaching model self-study to self-summary to hands-on practice?” was used to assess students’ attitudes and gains from the FC approach during the learning process. The third question, “How do you feel about the teaching model in which the teacher shows and then performs the hands-on?” was designed to obtain students’ feedback on the traditional teaching method in the implant dentistry hands-on course.

**Table 1:** Distribution of responses to the satisfactory questionnaire

Satisfaction Questionnaire		A	B	C	D	E
1.What is your overall feeling about this practical implant class? A.It’s great. I love it. B.Not bad. C.Not so good. D.I do not like it.	Group A Group B	91.67% 97.5%	8.33% 2.5%			
2.What do you think about the teaching model “self-study to self-summary to hands-on practice? A.Promote your own pre-reading and reflection. B.Video learning materials can be studied repeatedly. C.Video study materials are more convenient for learning. D.Stimulate self-learning and increase confidence. E.I don’t like pre-class self-study and self-summarize, I prefer to listen to the teacher’s lecture.	Group A	77.78%	91.67%	83.33%	22.22%	5.56%
3. How do you feel about the teaching model where the teacher shows and then performs the hands-on? A. The teacher’s explanations made more understandable. B. The teacher’s explanation made me pay more attention to the details of the operation. C. The teacher’s instructions were so clear that I mastered them in one time. D. You can only see it once. If you miss it, you can’t go back.	Group B	82.5%	85%	60%	30%	

### Statistical analysis

SPSS 26 statistical software was used to analyze the data. The Shapiro-Wilk test determined whether the study subjects conformed to the normal distribution, and a t-test analyzed the results. A statistically significant difference was indicated by *P* < 0.05.

## Results

### Participants attendance

A total of 80 students were included in the study. Of the 80 students who participated in the implantation hands-on class, one model in the control group was discarded because of operational mishandling. A total of 76 students provided feedback via the satisfactory questionnaire: 36 students in the FC group and 40 in the control group.

### Implant linear deviations

**Fig. 2:**
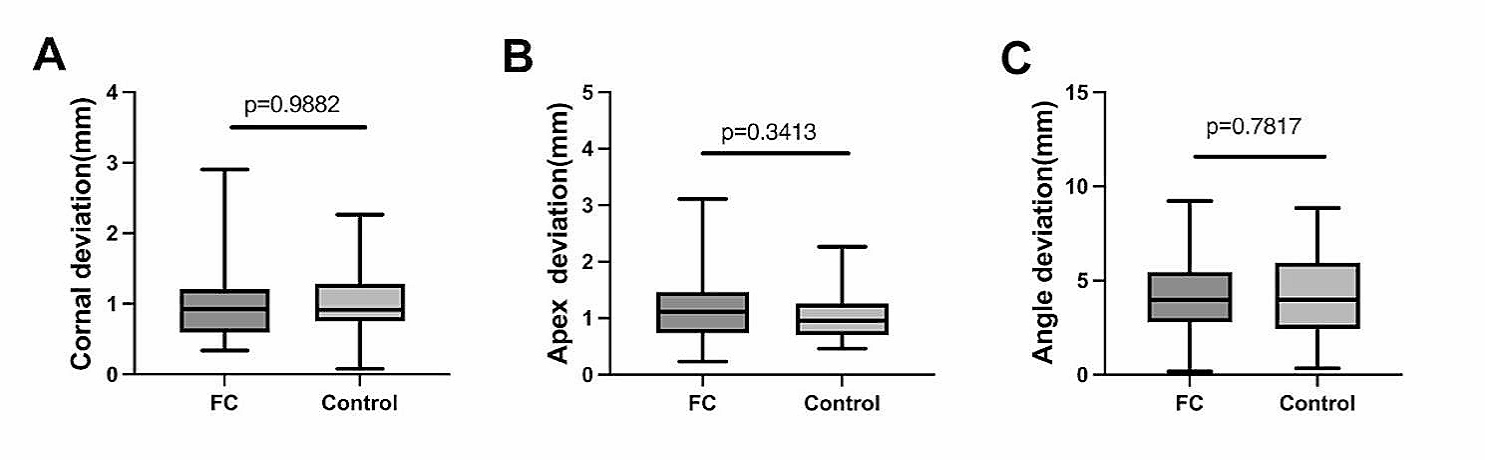
CBCT analysis of implant linear deviations between the two groups. Implant linear deviation between the FC and the control group at the coronal implant region (**A**), at the apex implant region (**B**), and at the angular level (**C**)

To analyze the accuracy of implant placement, CBCT of students’ models was used to match the standard model which was placed by the teacher under dynamic navigation (Fig. 2). The mean linear deviation at the coronal implant region was 1.016 mm in the FC group and 1.018 mm in the control group respectively (*p* = 0.9882). The mean linear deviation at the apex was 1.173 mm in the FC group and 1.058 mm in the control group, respectively (*p* = 0.3413). The mean angular deviation was 4.321 degrees in the FC group and 4.183 degrees in the control group, respectively (*p* = 0.7817).

### Prosthetic deviations

**Fig. 3:**
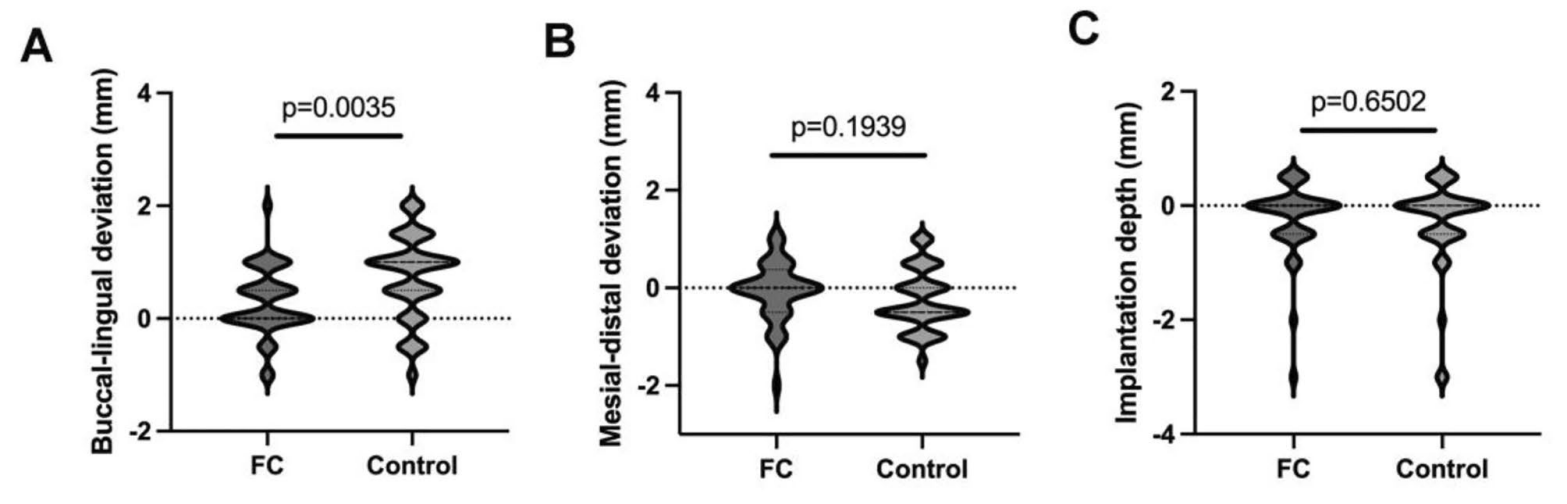
Semiquantitative restoration deviation measurement by the novel gauge between the FC and the control group. The deviation at buccal-lingual direction (**A**), Mesial-distal direction (**B**), and coronal-apex direction (**C**). (Direction illustration: + referring to buccal, - referring to lingual in figure **A**; (+ referring to mesial, - referring to distal in figure **B**; + referring to coronal, - referring to apex in figure **C**)

Apart from using CBCT to examine each group’s implant linear deviation, a novel restoration-level deviation gauge was utilized to check the students’ implant prosthetic-level accuracy (Fig. 1). The results showed that a significant buccal deviation was observed in the control group compared to the FC group (buccal deviation mean: 0.7436 mm v.s.0.2875 mm, *p* = 0.0035) (Fig. 3A). However, no statistical difference was observed between the FC group (mean = 0.0625 mm distal deviation) and the control group (mean = 0.2436 mm distal deviation) for mesial-distal deviation (*p* = 0.1939) (Fig. 3B). The difference in implantation depth in the coronal-apical direction between the two groups was the minimum (*p* = 0.6502) (Fig. 3C).

**Fig. 4:**
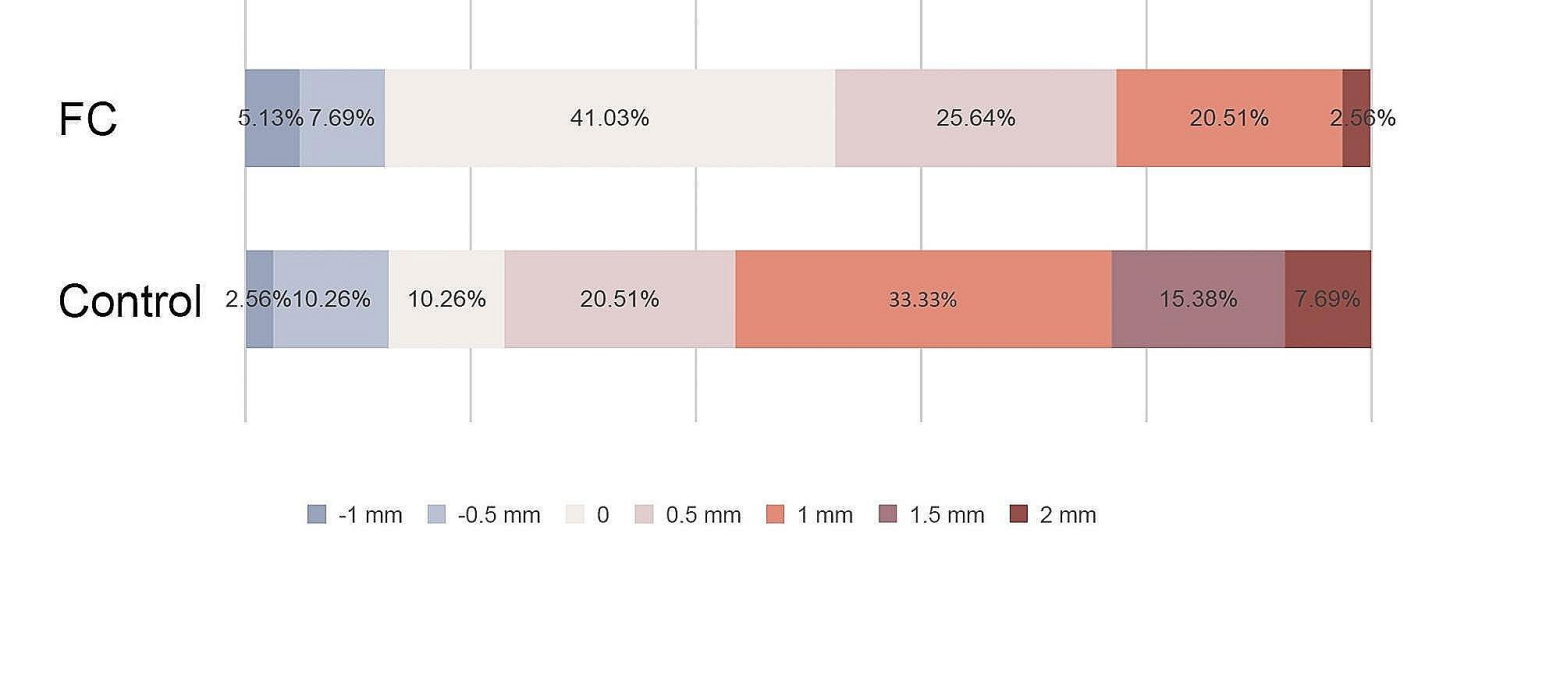
The percentage distribution of restoration linear deviation at the buccal-lingual direction (+ referring to buccal deviation, - referring to lingual deviation)

Considering the measurement discernible to the naked eye, the restoration deviation accuracy was graded at every 0.5 mm. The percentage distribution of the restoration linear deviation was also analyzed. In the FC group, 74.36% of the participants placed implants within 0.5 mm of buccal-to-lingual deviations, but only 41.03% of the control group (29 students) did the same (Fig. 4). A total of 41.03% of the FC group (16 students) achieved implant placement with no visually visible discrepancy, but only 10.26% of the control group (4 students) achieved this (Fig. 4). Students in the control group pretended to place implants more buccally than those in the FC group. Thus, the FC approach could significantly improve students’ control of buccal deviation at the restoration level.

**Fig. 5:**
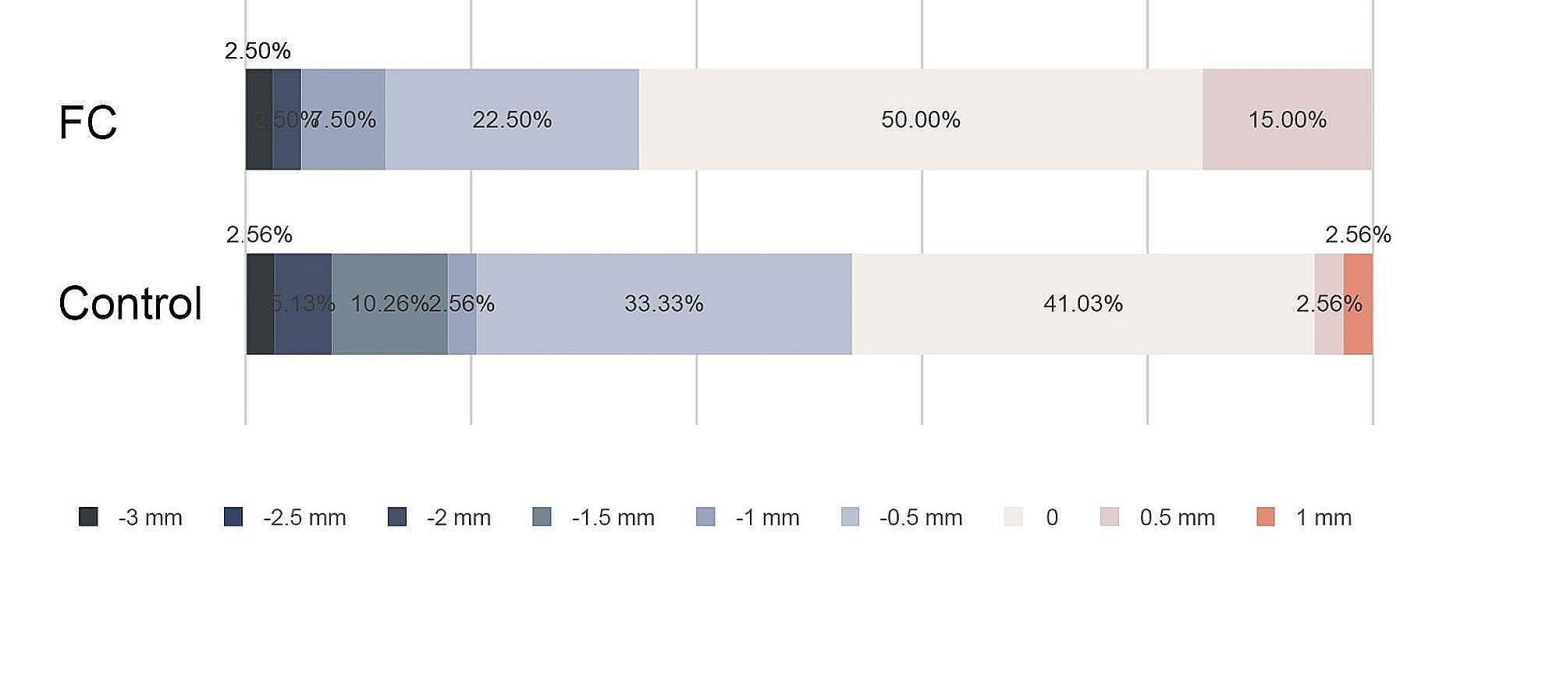
The percentage distribution of restoration linear deviation at the mesial-distal direction (+ referring to mesial deviation, - referring to distal deviation)

As for the mesial-distal of the restoration linear deviation, the distal deviation was greater than the mesial deviation in both groups (Fig. 5). In the FC group, 87.5% of students (34 students) placed implants within 0.5 mm of mesial-to-distal deviations, and in the control group, 74.39% of the students (29 students) did the same (Fig. 5). The implant placement deviation was greater than 1 mm for 20.51% of the control group and only 5% for the FC group (Fig. 5).

**Fig. 6:**
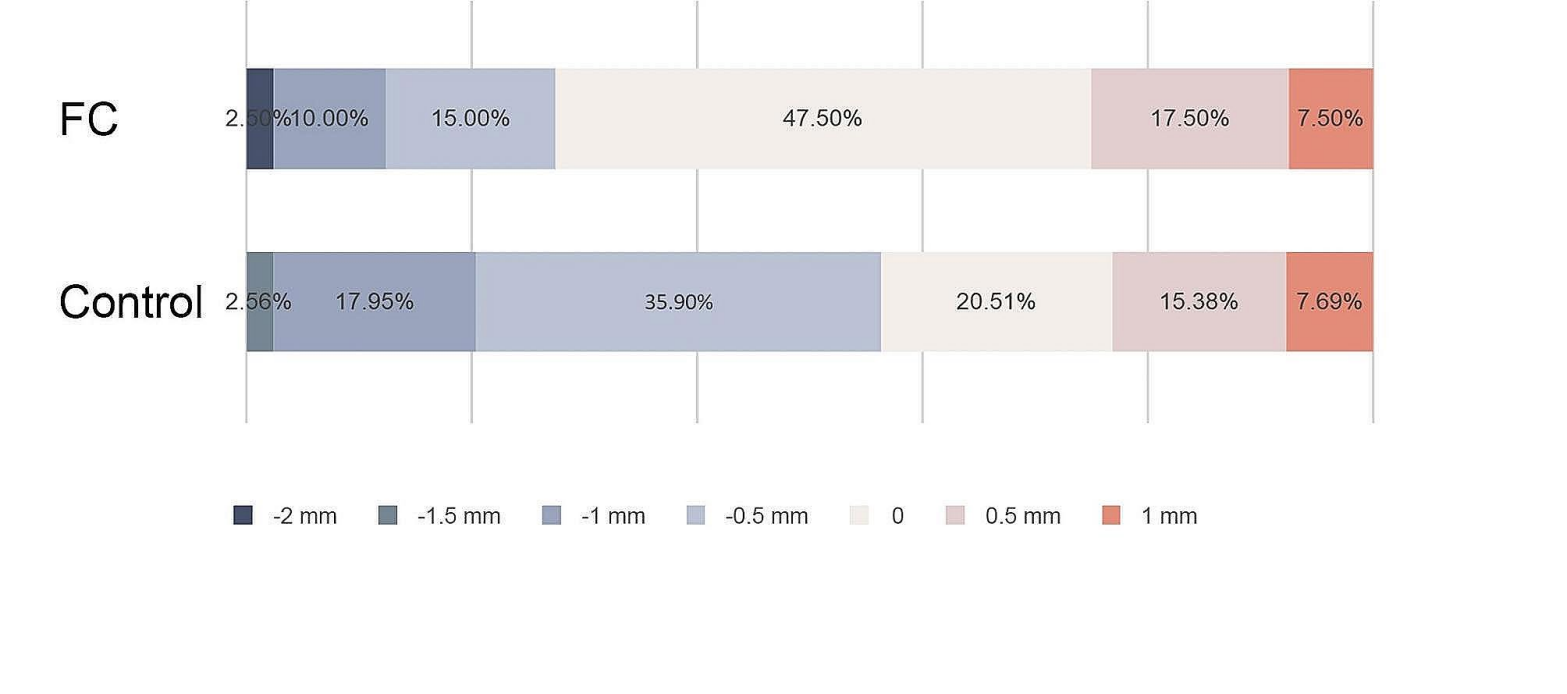
The percentage distribution of restoration linear deviation at the coronal-apex direction (+ referring to coronal deviation, - referring to apex deviation)

A total of 47.5% of students in the FC group were able to control the implant platform flush with the alveolar crest level, compared to 20.51% of students in the control group. Interestingly, the students preferred to place the implant 0.5 mm beneath the alveolar crest level in the control group (Fig. 6).

### Satisfaction questionnaire results

According to the satisfaction survey (Table 1), 91.67% of the students in the FC group felt positive about the implant hands-on course, while 97.5% of the students in the control group agreed. A total of 77.78% of students in the FC group thought that this teaching method could promote their pre-reading and reflection, 91.67% of students indicated videos can be studied repeatedly, 83.33% found that videos were more convenient for learning, 22.22% thought that the flipped classroom approach could stimulate self-learning and enhance confidence, and only 5.56% of the students did not like pre-class self-study (Table 1). In the control group, over 80% of students noted that their teacher’s explanation can make the class clearer, and give more details of the operation, 85% considered the teacher’s explanations made more understandable, and 60% stated they could master the operation the first time because of the teacher’s clear instructions. However, 30% of students indicated that the teacher’s one-time demonstration could not be repeated for reviews if they missed it (Table 1).

## Discussion

The core features of the FC approach include students’ previewing teaching content (e.g., a pre-recorded lecture, PowerPoint presentations, websites, and bibliographic references, et al.) in advance, and the teacher was aware of students’ understanding and learning status during the flipped learning activities [9]. Videos have been frequently validated for dentistry teaching when a practical component is essential [3]. Accordingly, our study focused on the application of instructional videos in the FC approach to implant dentistry hands-on teaching.

The status of implant dentistry education among undergraduates from 34 institutions in 18 European countries was assessed. The average amount of time assigned to implant dentistry was 36 h, with a range of 3 to 120 h. All the institutions provided theoretical courses, but only 65% offered pre-clinical training. Half of the schools permitted students to assist in implant surgery and prosthetic treatment sessions, but less than one-third of the schools allowed students to treat clinical patients [3]. It has become a mandatory curriculum at Wuhan University for postgraduate students since the year 2016, and for undergraduate students since the year 2021. In senior undergraduate implant teaching (the fourth or fifth year), students are arranged for implant placement hands-on classes on simulation models before attending the clinic. The traditional teaching mode is widely used for dental implantology practice in China. The advantage of this teaching model is that the teacher’s on-site demonstration can bring a deep impression, while the disadvantage is that it leads to a lack of independent learning and self-exploration of the students. The Association for Dental Education in Europe (ADEE) proposed a consensus on guidelines for the teaching and assessment of implant dentistry at the undergraduate level. The consensus concerning implant surgical procedures demonstrated that the undergraduate should handle the surgical principles and major techniques for the surgical placement of dental implants [18]. Thus, pre-clinical training on the simulation model has a positive influence on the attitude toward implant dentistry surgical knowledge [19]. The FC teaching approach usually applied to theory teaching, may have a different effect when applied to dental implant practice courses.

Clinical practice skill requires tight integration of theoretical knowledge with hand-eye-brain coordination in clinical realities [20]. The FC model could serve as a student-centered method in clinical hands-on skills. In our study, the analysis of the results of implant deviation via CBCT did not indicate a statistical difference between the FC and the control group. However, the FC group took less time than the control group (1.5 h vs. 2.5 h) to reach a similar learning goal. Research exploring the FC approach in clinical practical skill teaching is scant. A recent study emphasized the FC approach resulted in better student performance in five areas of clinical skills: intravenous catheterization (IV), IV blood collection, blood pressure measurement from the brachial artery, intramuscular injection into the ventrogluteal region, and urinary catheterization in women [20]. Another study exploring third-year undergraduate dental students fabricating orthodontic wire-bending skills also compared FC with the live demonstration (LD) approach, and concluded that the FC outperformed the LD approach in fostering personalized learning and improving the efficacy of physical class time, but LD was more advantageous than FC in allowing immediate question and answer [21].

To more accurately and quickly assess students’ practical deviations, a novel deviation-measuring gauge was invented that could offer more timely feedback to the students and timely correction of errors compared with a periodontal probe (Fig. 1). This unique gauge is made of resin, and fixed on the neighboring teeth on either side. To facilitate observation and accurate reading, an evaluation scale was provided on each side wall, and a conspicuous red sign was stamped on the center. Once the implant is connected to the transfer bar, the implantation deviation can be measured on the restoration. With this gauge, students can achieve timely self-evaluation in the classroom, analyze the reasons for mistakes, self-summarize, and make practical skill improvements. Thus, teachers can accurately analyze the implant position, depth, and angular deviation, and correspondingly help students improve their knowledge of implantation technology. Rather than taking a CBCT after class, teachers can use this gauge to assist in judging implant placement accuracy based on restoration-level deviation. In our study, a significant difference was found when we penetrated the implant out to the position of the crown to judge the restoration-level deviation. Regarding this phenomenon, we speculate that these differences occurred because students could view the video several times, focus on more surgical details, and learn at their own pace (by pausing, rewinding, and replaying the video), unlike with the one-time on-the-spot surgery demonstration.

Most of the students found the two teaching methods interesting because oral implantology hands-on practice was completely new to them. The majority of the students (91.67%) in the FC group responded “It’s great. I love it”, and a higher percentage in the control group (97.5%) chose this option. These responses may be due to the FC approach requiring students to think independently and interact actively. A total of 91.67% of students agreed that the FC teaching method could allow the instructional video to be repeated, and 83.33% of students thought these repeats could bring more convenience. These results coincide with those obtained in the pediatric dentistry course, in which students stated the video was a very useful tool [9]. Faraone et al. implemented a blended curriculum model on complete denture prosthodontics, in which audio-visual and written materials were provided at the beginning of the semester. Students on this course were satisfied with comments like “This course allowed me to move at my own pace. I was having difficulty with a few procedures so I watched and re-watched the videos and reviewed the lectures at home and then went back to the school to master those steps. The asynchronous structure of this course was very conducive to my productivity and progress” [11]. Pre-class video lectures can be accessed at any time and as often as students desire. Students also highly appreciated the use of small-group discussion-based activities in the FC face-to-face sessions because these sessions helped increase their interest in subject and their motivation to learn.

The FC teaching approach can improve students’ engagement inside and outside the classroom, and cultivate critical thinking and problem-solving abilities through small-group discussion and the instructor’s assistance [9]. With the traditional lecture method, students can only focus on teaching for approximately 15 min and only 20% retained the content. However, the FC teaching approach engages students in the classroom with various activities related to the previous homework assignment so that students can concentrate for a longer time and knowledge can be better retained because of active engagement [22]. Introducing the FC teaching approach to the implant dentistry practical course not only allows students to get more opportunities for practical exercise and skills within the limited class time, but also stimulates the students’ subjective initiative, which creates positive feedback. Precise surgical operation and subjective initiative are both significant abilities for a qualified surgeon, and the FC teaching method applied to the implant dentistry practical course exactly focuses on the development of these two aspects of the student’s ability. In addition, this study will provide an important reference for the reform of teaching methodology in oral implant practice and expand the application of the FC teaching approach.

Even though the FC teaching approach was an effective model for learning implant dentistry through hands-on courses, it has limitations. Firstly, a well-organized video presentation and logistical planning required more input from teachers than traditional lectures. Secondly, it requires more classrooms to facilitate small-group discussions [3]. Thirdly, students were unwilling to work at home as is typically done in a traditional face-to-face class, and considered watching the pre-class videos to be a time [22]. Fourthly, the FC approach did not allow students to ask questions and receive immediate answers [21]. Finally, the online video demonstration is 2D, thus limiting the accurate representation of an actual 3D demonstration. As for the limitations of this study, on one hand, the summary debriefing of the practical course presented by each group tends to be homogeneous, lacking personalized viewpoints and extended discussion on the implementation of skills. On the other hand, we only compared the two teaching methods for the simulated placement of implants on the mandibular first molar. The problems in clinical are complex, and the results of this study cannot represent other complex situations Thus, more studies need to be applied to different degrees of difficulty in the classroom such as anterior aesthetic zone implantation and multi-tooth implantation to observe the effect FC approach in the dental implantology practice course.

## Conclusions

In conclusion, implementing the FC teaching approach in implant dentistry hands-on course improved learning efficiency more than the traditional approach in limited class time. Although students were less satisfied with the application of the FC teaching method to implant dentistry practical courses than the traditional teaching method due to an additional study burden and the requirement to think independently, the majority of students appreciated pre-class video lectures. Therefore, the FC approach can be applied as in clinical practical skills training as an available teaching method.

## Data Availability

The authors declare that all the data and materials supporting the findings of this study are available within the article and are available from the corresponding author upon request.
